# Crystallographic and X-ray scattering study of RdfS, a recombination directionality factor from an integrative and conjugative element

**DOI:** 10.1107/S2059798322008579

**Published:** 2022-09-27

**Authors:** Callum J. Verdonk, Andrew C. Marshall, Joshua P. Ramsay, Charles S. Bond

**Affiliations:** a School of Molecular Sciences, University of Western Australia, Perth, Western Australia 6009, Australia; b Curtin Health Innovation Research Institute and Curtin Medical School, Curtin University, Perth, Western Australia 6102, Australia; c Marshall Centre for Infectious Disease, Research and Training, School of Biomedical Sciences, University of Western Australia, Perth, Western Australia 6009, Australia; University of Cambridge, United Kingdom

**Keywords:** RdfS, recombination directionality factor, *Mesorhizobium*, noncrystallographic symmetry, winged helix–turn–helix domain, DNA binding, excisionase

## Abstract

The recombination directionality factors from *Mesorhizobium* spp. (RdfS) are involved in regulating the excision and transfer of integrative and conjugative elements. Here, solution small-angle X-ray scattering, and crystallization and preliminary structure solution of RdfS from *Mesorhizobium japonicum* R7A are presented. RdfS crystallizes in space group *P*2_1_2_1_2_1_, with evidence of eightfold rotational crystallographic/noncrystallographic symmetry. Initial structure determination by molecular replacement using *ab initio* models yielded a partial model (three molecules), which was completed after manual inspection revealed unmodelled electron density. The finalized crystal structure of RdfS reveals a head-to-tail polymer forming left-handed superhelices with large solvent channels. Additionally, RdfS has significant disorder in the C-terminal region of the protein, which is supported by the solution scattering data and the crystal structure. The steps taken to finalize structure determination, as well as the scattering and crystallographic characteristics of RdfS, are discussed.

## Abstract 2

The X-ray crystallographic structure of RdfS reveals molecular superhelical polymers in the crystal.

## 1. Introduction

Integrative and conjugative elements (ICEs) are chromo­somally integrating mobile genetic elements that transfer between bacteria using conjugation. Prior to conjugation, ICEs must excise from the bacterial chromosome, a process facilitated by an ICE-encoded site-specific recombinase (also known as an integrase) and an additional protein called a recombination directionality factor (RDF, also know as excisionase; Groth & Calos, 2004 ▸; Lewis & Hatfull, 2001 ▸; Ramsay *et al.*, 2006 ▸). The integrase binds regions of DNA called attachment sites (*att* sites), which contain a catalytic ‘core’ site where strand exchange occurs and conserved flanking regions called ‘arm’ or ‘P’ sites that orchestrate the structural organization of the nucleoprotein complex (Radman-Livaja *et al.*, 2005 ▸). RDFs are often winged-helix–turn–helix domain proteins that also bind DNA within *att* sites (Sam *et al.*, 2004 ▸; Lewis & Hatfull, 2001 ▸). DNA binding by the RDF alters the recombinase–DNA nucleoprotein complex and often bends DNA to switch the favoured direction of recombination towards ICE excision.

ICE*Ml*Sym^R7A^ is a 502 kb ICE encoded by *Mesorhizobium japonicum* R7A which confers on its host the ability to fix nitrogen and form symbiosis with leguminous plants of the *Lotus* genus (Sullivan & Ronson, 1998 ▸; Sullivan *et al.*, 2002 ▸). The *att* sites *attL* and *attR* flank the ICE (Sullivan & Ronson, 1998 ▸). Integration requires the integrase, IntS, a tyrosine recombinase that belongs to the P4 integrase family (Sullivan & Ronson, 1998 ▸; Esposito & Scocca, 1997 ▸; Ramsay *et al.*, 2006 ▸; Verdonk *et al.*, 2019 ▸). When excised, the *att* sites are recombined, producing *attP* on the extrachromosomal circularized ICE*Ml*Sym^R7A^ and *attB* within the bacterial chromosome. Excision of ICE*Ml*Sym^R7A^ requires IntS and the RDF (also known as excisionase) RdfS, which when expressed stimulates ICE*Ml*Sym^R7A^ excision and the concomitant formation of *attP* and *attB* (Ramsay *et al.*, 2006 ▸). The expression of *rdfS* in ICE*Ml*Sym^R7A^ is stimulated by quorum sensing (Ramsay *et al.*, 2015 ▸, 2021 ▸).

Secondary-structure prediction of RdfS proteins found in *Mesorhizobium* spp. suggest that they are members of the MerR superfamily of winged-helix–turn–helix (wHTH) DNA-binding proteins (Lewis & Hatfull, 2001 ▸; Haskett *et al.*, 2018 ▸). Deletion of *rdfS* from the R7A chromosome abolishes the excision of ICE*Ml*Sym^R7A^, and overexpression of *rdfS* results in loss of ICE*Ml*Sym^R7A^ from the cell. An intact *rdfS* is also required for conjugation (Ramsay *et al.*, 2006 ▸; Verdonk *et al.*, 2019 ▸). RdfS homologues on related ICEs also act as transcriptional activators (Haskett *et al.*, 2016 ▸, 2017 ▸, 2018 ▸). Some RDFs, such as those of the *cox* family in phages, have also been shown to act as transcriptional regulators (Lewis & Hatfull, 2001 ▸; Lundqvist & Bertani, 1984 ▸; Dodd *et al.*, 1990 ▸; Saha *et al.*, 1987 ▸; Esposito & Scocca, 1997 ▸; Ahlgren-Berg *et al.*, 2009 ▸). RdfS is highly conserved across diverse *Mesorhizobium* spp. that carry ICEs (Colombi *et al.*, 2021 ▸) and also among plasmids, suggesting additional roles of RDFs in transfer aside from those involved in recombination (Verdonk *et al.*, 2019 ▸; Ramsay *et al.*, 2006 ▸). There are currently no experimental structures of RdfS homologues (from meso­rhizobia or otherwise) within the Protein Data Bank (PDB).

In this study, we cloned and overexpressed RdfS from *M. japonicum* R7A and determined its X-ray crystal structure to 2.45 Å resolution. We also demonstrate a solution scattering model of monomeric RdfS using small-angle X-ray scattering.

## 2. Materials and methods

### 2.1. Expression and purification of RdfS

The *rdfS* gene (*msi109*) encoding the 89-residue protein (UniProt ID Q7AL96) was amplified from *M. japonicum* R7A (GenBank accession CP051772) genomic DNA using PCR (5′-ATAT**CCATGG**ACGACGAAAACGACCGC-3′ and 5′-ATAT**GGATCC**TTATCATGAGCGGGCTCCCTCG-3′; NcoI and BamHI sites in bold). The PCR product was cloned into the NcoI/BamHI sites of the pETM11 expression vector (European Molecular Biology Laboratory; Dümmler *et al.*, 2005 ▸) using T4 DNA ligase (New England Biolabs; NEB) as per the manufacturer’s instructions. Confirmation of the insert was performed using PCR and subsequent Sanger sequencing (Australian Genome Research Facility). The plasmid was transformed into electrocompetent *Escherichia coli* NiCo21(DE3) cells (NEB) using electroporation and was selected for on lysogeny broth (LB) agar medium containing kanamycin (50 µg ml^−1^). Single colonies were inoculated into 5 ml LB (50 µg ml^−1^ kanamycin) and incubated for 16 h at 310 K at 160 rev min^−1^. The 5 ml culture was used to inoculate 1 l nonselective LB in a 5 l conical flask incubated at 310 K at 160 rev min^−1^ until an optical density (600 nm) of ∼0.5 was reached. The cells were then induced using isopropyl β-d-1-thiogalactopyranoside at a final concentration of 0.1 m*M* and grown for an additional 16 h at 293 K with shaking at 160 rev min^−1^. The cells were harvested at 4°C for 45 min at 20 000*g*. The cell pellets were resuspended in wash buffer [80 m*M* NaH_2_PO_4_, 500 m*M* NaCl, 80 m*M* imidazole, 5%(*v*/*v*) glycerol; pH 7.4] before being lysed using an Emulsiflex C5 high-pressure homogenizer (Avestin).

The lysate was centrifugated at 24 000*g* for 45 min at 4°C and the clarified soluble lysate was filtered using a 0.22 µm filter before being loaded onto a 5 ml HisTrap column (Cytiva) using an EP-1 peristaltic pump (Bio-Rad). Hexahistidine-tagged RdfS (6H-RdfS) was eluted from the column using a linearly increasing concentration of elution buffer [80 m*M* NaH_2_PO_4_, 500 m*M* NaCl, 850 m*M* imidazole, 5%(*v*/*v*) glycerol; pH 7.4] across a total of ten column volumes (50 ml) on an ÄKTApure chromatography system (GE Healthcare).

Fractions containing 6H-RdfS as determined by a 280 nm UV trace were pooled and diluted to approximately 2 mg ml^−1^ in TEV digestion buffer [50 m*M* Tris–HCl pH 7.5, 300 m*M* NaCl, 1 m*M* EDTA, 5%(*v*/*v*) glycerol, 1 m*M* DTT]. In-house-prepared uncleavable 6H-tagged TEV protease (2.5 mg ml^−1^) was added to 6H-RdfS at a ratio of 1:10(*v*:*v*) TEV:6H-RdfS before dialysing overnight in TEV digestion buffer at ambient temperature with gentle agitation. Post-digestion, the 6H-RdfS/TEV mixture was briefly centrifuged and filtered using a 0.22 µm filter before being reapplied onto the 5 ml HisTrap column pre-equilibrated with TEV digestion buffer. The flowthrough containing cleaved RdfS was pooled and concentrated to a final volume of 5 ml using a 3 kDa 15 ml centrifugal filter (Amicon) before being loaded onto a HiLoad 16/600 Superdex 200 column (GE Healthcare) pre-equilibrated with SEC buffer [50 m*M* Tris–HCl, 300 m*M* NaCl, 5%(*v*/*v*) glycerol; pH 7.4].

Peak fractions were pooled, concentrated and stored at room temperature for immediate use or flash-frozen in liquid nitrogen for long-term storage at −80°C.

### 2.2. Small-angle X-ray scattering

Small-angle X-ray scattering (SAXS) data were collected on the SAXS/WAXS beamline at the Australian Synchrotron (Kirby *et al.*, 2013 ▸) with continuous data collection using a PILATUS 1M detector (Broennimann *et al.*, 2006 ▸). All 6H-RdfS SAXS data were collected using size-exclusion chromatography-coupled synchrotron SAXS (SEC-SY-SAXS; Gully *et al.*, 2015 ▸) with a Superdex 200 Increase 5/150 GL column (GE Healthcare) controlled by a Shimadzu HPLC system. All experiments were performed in SAXS buffer [150 m*M* Tris–HCl, 300 m*M* NaCl, 5%(*v*/*v*) glycerol; pH 7.4]. SAXS data-collection and analysis statistics are given in Table 1 ▸. Scattering data were background-corrected using linear interpolation of the background from averaged frames using *scatterBrain* (Stephen Mudie, Australian Synchrotron). The intensity at zero [*I*(0)], Guinier range and radius of gyration (*R*_g_) were determined using the *ATSAS* package (Manalastas-Cantos *et al.*, 2021 ▸) with *PRIMUS* (Konarev *et al.*, 2003 ▸) and *GNOM* (Semenyuk & Svergun, 1991 ▸). SAXS *ab initio* modelling was performed using *DAMMIN* (Svergun, 1999 ▸) and *DAMMIF* (Franke & Svergun, 2009 ▸). Refinement of *ab initio* models was performed by the *DAMAVER* suite (Volkov & Svergun, 2003 ▸) using 11 individual *DAMMIN* models, and the solution scattering of 6H-RdfS was compared with the finalized X-ray structure using *CORAL* (Manalastas-Cantos *et al.*, 2021 ▸) and *EOM* (Tria *et al.*, 2015 ▸; Bernadó *et al.*, 2007 ▸). The final SAXS data have been deposited in the SASBDB under entry SASDPK4.

### 2.3. Crystallization

For crystallization, the protein tags were removed. Crystallization trials were explored using a variety of sparse-matrix screens: Index HT (Hampton Research), Crystal Screen HT (Hampton Research), JCSG-*plus* (Molecular Dimensions; Newman *et al.*, 2005 ▸), the LMB Crystallization Screen (Molecular Dimensions) and ProPlex (Molecular Dimensions). Each screen was set up in sitting-drop vapour-diffusion format in a three-drop 96-well ARI LVR Intelli-Plate (Hampton Research) at 293 ± 0.5 K using an Art Robbins Phoenix robot. Each 300 nl drop consisted of protein solution (9 mg ml^−1^) and reservoir solution in a 1:1, 1:2 or 2:1 ratio equilibrated against 80 µl reservoir solution. While crystallization was observed in dozens of conditions across the entire plate, larger needle-shaped crystals that formed in condition No. 38 of the ProPlex screen [0.1 *M* MES pH 6.5, 10%(*w*/*v*) PEG 5000 MME, 12%(*v*/*v*) 1-propanol] appeared to be the most promising. Further optimization attempts varying the pH and the precipitant concentration in hanging drops in a 24-well VDX plate (Hampton Research) produced similar crystals with a larger size (∼50 × 1000 µm) which diffracted poorly (>10 Å). One variation of the initial condition was selected from a modified protocol of the Additive Screen matrix (Hampton Research) set up across four hanging-drop 24-well VDX plates, with each drop consisting of 2.5 µl protein (8.8 mg ml^−1^), 2 µl crystallization condition [0.1 *M* MES pH 6.5, 8%(*w*/*v*) PEG 5000 MME, 10%(*v*/*v*) 1-propanol] and 0.5 µl additive condition equilibrated over a reservoir containing 100 µl of only the additive condition. This unusual setup was the result of an error which proved to be productive. In the presence of additive No. 23 (1 *M* sodium citrate tribasic dihydrate) RdfS formed large, multi-nuclear crystals of >1 mm in length. Replicate trials set up with identical conditions were conducted with four drops per well in a 24-well VDX hanging-drop tray (a total of 96 identical drops), with three individual drops producing plate-like rod crystal clusters [the final conditions in the drop were 0.05 *M* MES pH 6.5, 4%(*w*/*v*) PEG 5000 MME, 5%(*v*/*v*) 1-propanol, 0.1 *M* sodium citrate with RdfS protein at 4.3 mg ml^−1^]. Crystal clusters were harvested and transferred to a cryoprotectant condition [0.08 *M* MES pH 6.5, 6.4%(*w*/*v*) PEG 5000 MME, 8%(*v*/*v*) 1-propanol, 20%(*v*/*v*) ethylene glycol] for 2 min. Micro-Tools (Hampton Research) were used to separate individual crystals from the clusters before harvesting and flash-cooling in liquid nitrogen (Haas & Rossmann, 1970 ▸; Henderson, 1990 ▸). Diffraction experiments were carried out on the MX2 beamline at the Australian Synchrotron, Melbourne, Victoria, Australia (Aragão *et al.*, 2018 ▸) using remote access via the *Blu-Ice* software (McPhillips *et al.*, 2002 ▸). The best data set was collected at 13.0 keV (λ = 0.953 Å) with a crystal-to-detector distance of 380 mm (2.49 Å at the detector top edge).

### 2.4. Diffraction data collection, processing and refinement

Data-collection and processing statistics are presented in Table 2 ▸. Data were collected at 100 K using a Dectris EIGER 16M detector (Casanas *et al.*, 2016 ▸), with a total rotation range of 180° and an exposure time of 36 s at 60% attenuation of the beam. Data were integrated and reduced with *XDS* (Kabsch, 2010 ▸) and were scaled, merged and truncated in space group *P*2_1_2_1_2_1_ using *AIMLESS* (Evans & Murshudov, 2013 ▸). Solvent-content analysis was performed using the Matthews coefficient (Matthews, 1968 ▸). The self-rotation function was calculated with *MOLREP* (Vagin & Teplyakov, 2010 ▸) using data between 42.87 and 2.65 Å resolution with an integration radius of 33.96 Å. Molecular-replacement calculations were carried out in space group *P*2_1_2_1_2_1_ using *MOLREP* (Vagin & Teplyakov, 2010 ▸) from the *CCP*4 suite (Winn *et al.*, 2011 ▸) with five individual versions of *ab initio* models of truncated RdfS generated from *ColabFold* (Mirdita *et al.*, 2022 ▸) using the modelling algorithms from *AlphaFold*2 (Jumper *et al.*, 2021 ▸; henceforth referred to as *AlphaFold*) and the sequence-alignment algorithms from *HHsearch* (Steinegger *et al.*, 2019 ▸) and *MMseqs*2 (Steinegger & Söding, 2017 ▸). The structure was initially refined with *REFMAC*5 (Murshudov *et al.*, 2011 ▸) using rigid-body refinement followed by restrained refinement with automatically generated local NCS restraints (Usón *et al.*, 1999 ▸). Refinement was completed with *phenix.refine* (Afonine *et al.*, 2012 ▸). NCS restraints were removed for the final stages of refinement. For refinement, 4.7% of reflections were used for cross-validation (Brünger, 1992 ▸). The fit of the structure to the electron density was manipulated with *Coot* (Emsley *et al.*, 2010 ▸). The coordinates and structure factors of the final, fully refined model (validated by *MolProbity*; Chen *et al.*, 2010 ▸) have been deposited in the Protein Data Bank (Berman *et al.*, 2000 ▸; PDB entry 8dgl) and the accompanying structural and biochemical interpretations will be reported elsewhere.

## 3. Results and discussion

### 3.1. Solution X-ray scattering of monomeric 6H-RdfS

**Figure 1:**
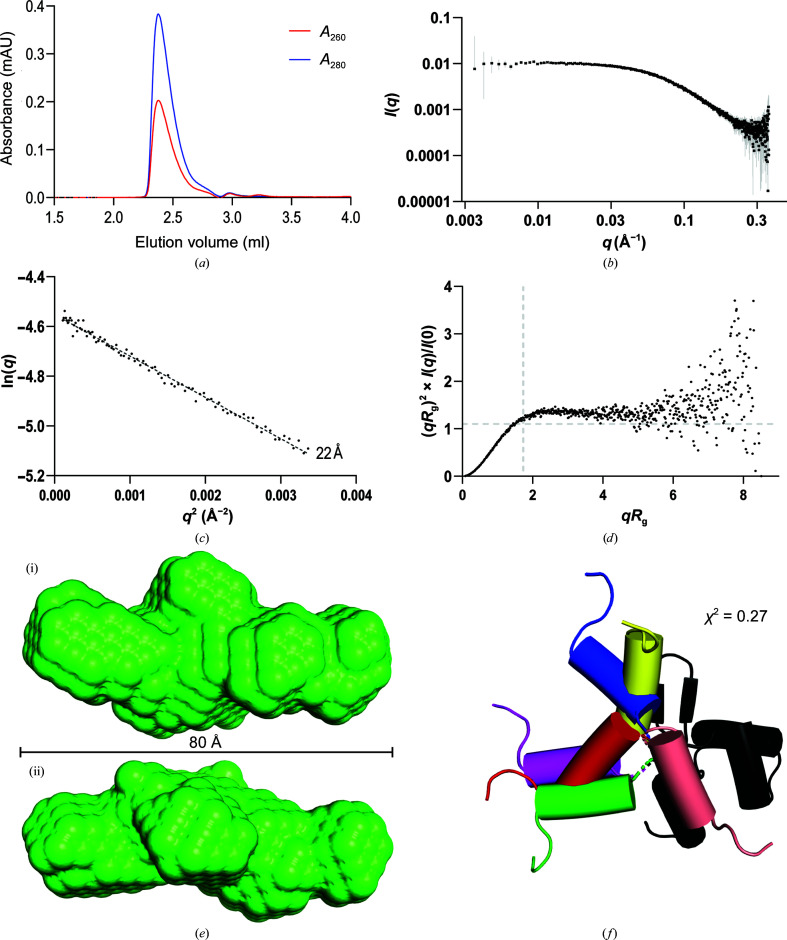
Characterization of 6H-RdfS in solution using small-angle X-ray scattering. (*a*) Size-exclusion chromatography profile of the 6H-RdfS monomer during the SAXS experiment. The absorbance at 260 and 280 nm is plotted against the elution volume from the Superdex 200 5/150 GL gel-filtration column. (*b*) Small-angle X-ray scattering profile of 6H-RdfS [log(*I*) versus log *q*]. (*c*) Guinier analysis of 6H-RdfS. *R*_g_ is shown adjacent to the plot line. (*d*) Normalized (dimensionless) Kratky plot of 6H-RdfS. The peak 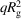 is shown as a dashed line. (*e*) *Ab initio* *DAMFILT*-filtered bead model (based on 11 *DAMMIN* models) representing the elongated 6H-RdfS envelope in (i) elongated and (ii) perpendicular views. The mean iterative closest point (ICP; Besl & McKay, 1992 ▸) metric was 14.7 (±2.5) for all 11 *DAMMIN* models. (*f*) *EOM* ensemble of six RdfS cartoon models fitted into the 6H-RdfS SAXS scattering data, with the wHTH domain (black) rigid and the N-terminal helix (coloured) disordered. The various colours demonstrate the flexibility of the N-terminal helix as fitted to the scattering data (red, green, yellow, blue, purple and pink).

Recombinant 6H-RdfS could be expressed and purified with a high yield (∼20 mg per litre of culture) suitable for biophysical analysis. Preliminary experiments were ambiguous with respect to the oligomerization state of RdfS in solution, so we used analytical SEC and SEC-SY-SAXS of 6H-RdfS to explore this in detail. 6H-RdfS elutes from the SEC column as a single peak with a long trailing edge and with an *A*_260 nm_:*A*_280 nm_ ratio of 0.5, indicating pure protein (Fig. 1 ▸ *a*). SAXS analysis of the peak yielded an experimental scattering curve exhibiting properties of a monodisperse sample (Fig. 1 ▸). Guinier analysis provided a radius of gyration (*R*_g_) of 22 Å, and the pair-distribution function shows a maximal dimension (*D*_max_) of approximately 92 Å, both of which are reasonable for a 6H-RdfS monomer with a prolate shape. SAXS-derived molecular-mass estimates of 12 046 Da (using a *q*_max_ of 0.300 Å^−1^ and a *V* of 14 600 Å^3^; Fischer *et al.*, 2010 ▸) and 12 025 Da (Bayesian inference estimate; 96.22% credibility interval probability; Hajizadeh *et al.*, 2018 ▸) also compare favourably with the expected molecular mass of 12 172 Da (as calculated by *ProtParam*; Wilkins *et al.*, 1999 ▸). A dimensionless Kratky plot shows a peak slightly beyond 1.1 Å and a *qR*_g_ of 1.7 Å^−1^, suggesting that 6H-RdfS is elongated with significant flexibility, which is also supported by the increase in intensity at *qR*_g_ > 5 indicating particle flexibility/disorder (Fig. 1 ▸ *d*; Durand *et al.*, 2010 ▸; Bizien *et al.*, 2016 ▸; Trewhella *et al.*, 2017 ▸). Porod volume estimates of 230 Å suggest extremely large, elongated particles in solution. These data support the hypothesis that a substantial proportion of the 6H-RdfS monomer is disordered in solution, as it contains an N-terminal His_6_ tag, a flexible linker and a TEV cleavage site, and the final 31 residues of the native protein are highly disordered as predicted by *MobiDB-lite* (Necci *et al.*, 2021 ▸). Given the propensity of the 6H-RdfS molecules to be disordered in solution, models were generated to visualize flexibility using the X-ray structure as a template. A filtered scattering envelope shows an elongated (80 Å) particle (Fig. 1 ▸ *e*), which is slightly smaller than the idealized *D*_max_ calculated using the pair-distribution function (Supplementary Fig. S1). An ensemble of *EOM* models is shown in Fig. 1 ▸(*f*), highlighting the flexibility of the N-terminal region in solution with a reasonable fit (χ^2^ = 0.27; two degrees of freedom). Further models of the RdfS structure fitted into the scattering of 6H-RdfS, including the addition of dummy residues for the additional N- and C-terminal regions that are not represented in the X-ray structure, can be found in Supplementary Fig. S2.

### 3.2. Crystallization and X-ray data processing

RdfS crystallized readily across a broad range of screening conditions, with crystals consistently forming in approximately 17% of conditions in Index HT (Hampton Research), 10% of conditions in Crystal Screen HT (Hampton Research), 23% of conditions in JCSG-*plus* (Molecular Dimensions), 24% of conditions in the LMB Crystallization Screen (Molecular Dimensions) and 23% of conditions in ProPlex (Molecular Dimensions). Adjustments to the concentration of protein used and the volume of the drop appeared to have little impact on the number of crystals or of condition ‘hits’, but instead the volume ratio of protein sample (in SEC buffer) to crystallization condition used had the largest discernible impact on the size and number of crystals within a single drop. Crystal-forming conditions containing either a 1:1 ratio of protein to crystallization condition (present in approximately 87% of all crystals formed) or a 1:2 ratio (∼73%) yielded the most crystals (a 2:1 ratio only appeared in ∼43% of all crystal-forming conditions).

**Figure 2:**
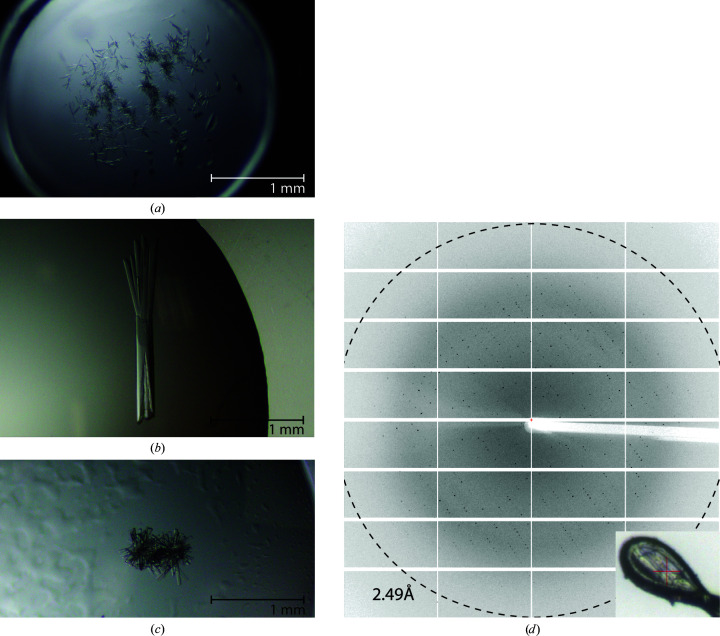
Crystallization and diffraction of RdfS. (*a*) An example crystal morphology from sparse-matrix crystallization screens, with a thin, rod-like crystal shape observed. (*b*) Initial additive trial multi-nuclear crystal as described in Section 3.2. (*c*) Final crystal morphology of the RdfS crystals used to generate the solved data set prior to separation with tools and cryoprotection. (*d*) Diffraction pattern from the first ten frames (1°) of data with a resolution indicator (dashed circle). An image of a crystal from the solved data set mounted in a loop is shown in the bottom right corner.

Hundreds of crystals from a variety of conditions were tested for diffraction with no success, which was perhaps not surprising given their thin, uneven needle-like habit (Fig. 2 ▸ *a*). Whilst these crystals varied in size, they typically formed very narrow (>10 µm width) highly elongated needles or prisms (Supplementary Fig. S3). No improvement in diffraction was seen with the MX2 mini beam (25 × 15 µm), compared with the larger MX1 beam (120 × 120 µm), for crystals with similar morphology. Condition No. 38 [0.1 *M* MES pH 6.5, 10%(*w*/*v*) PEG 5000 MME, 12%(*v*/*v*) 1-propanol] from the ProPlex screen yielded the most promising large (>500 µm) multi-nuclear crystals, which showed evidence of diffraction to 10 Å resolution (Supplementary Fig. S3). Optimization of the pH and the PEG and propanol concentrations [to 0.1 *M* MES pH 6.5, 8%(*w*/*v*) PEG 5000 MME, 10%(*v*/*v*) 1-propanol] was used to reproducibly obtain larger crystals.

To improve the diffraction properties, we screened additives and pursued a strategy capitalizing on an error where we screened for crystallization against reservoirs that contained *only* concentrated additive solution. Serendipitously, we found that this produced multiple crystal-forming conditions in initial trials with Hampton Research Additive Screen. Importantly, one of the conditions yielded a single large multi-nuclear crystal (>1 mm; Fig. 2 ▸ *b*) which diffracted to 4 Å resolution (data not shown). We replicated this experimental condition [0.05 *M* MES pH 6.5, 4%(*w*/*v*) PEG 5000 MME, 5%(*v*/*v*) 1-propanol, 0.1 *M* sodium citrate tribasic dehydrate with 4.3 mg ml^−1^ RdfS equilibrated against a reservoir consisting of 1 *M* sodium citrate tribasic dehydrate] 96 identical times, demonstrating low reproducibility (three successes out of 96 replicates; 3.1%), suggesting that variability in the process of setting up drops or uncontrolled nucleation events affected the outcome. Nevertheless, crystals from a single drop containing a typical large cluster of flat, rod-like crystals (Fig. 2 ▸ *c*) were harvested, cryoprotected in mother liquor supplemented with 20% ethylene glycol and flash-cooled for storage. We suspect that the effectiveness and the overall increase in diffraction quality of the crystals produced from our additive trials are the result of a ‘pseudo-salting-out’ technique. We assume that because of a decrease in water and 1-propanol in the drop over time via vapour diffusion due to the additive-only reservoir solution, idealized conditions fortuitously enabled RdfS to form crystals which diffracted well in a small percentage of replicate conditions.

RdfS crystals diffracted to 2.45 Å resolution on beamline MX2 at the Australian Synchrotron (Aragão *et al.*, 2018 ▸; Fig. 2 ▸ *d*). Data analysis clearly indicated space group *P*2_1_2_1_2_1_, with unit-cell parameters *a* = 35.25, *b* = 119.20, *c* = 123.40 Å (Table 2 ▸). Solvent-content analysis indicated six molecules per asymmetric unit to be most likely (46% probability), while a self-rotation function suggested that four or eight molecules per asymmetric unit were possible. With such high *Z*′ values and no experimental model of a close homologue, molecular replacement was expected to be challenging.

### 3.3. Molecular replacement

**Figure 3:**
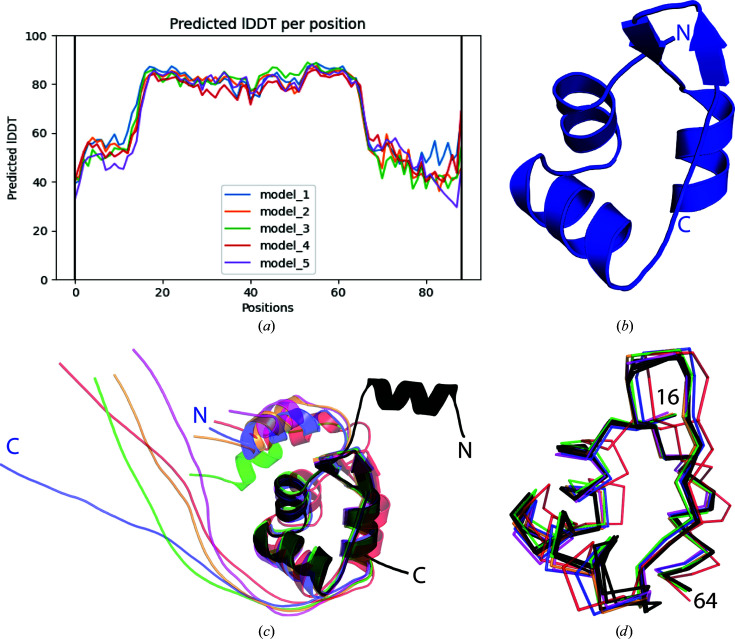
*Ab initio* models used in molecular replacement. (*a*) Predicted local distance difference test (pLDDT) residue position plot as generated by *ColabFold* showing the confidence in each residue (*y* axis) and the relative residue location (*x* axis) for the RdfS amino-acid sequence. The predicted wHTH domain and additional C-terminal helix (positions 16–64) have the highest confidence scores for each model. (*b*) The RdfS_16–64_ truncated model used in molecular replacement as described in Section 3.3. (*c*) Superposition of all full *AlphaFold*-generated models [coloured to match (*a*): blue, orange, green, red and purple] with the finalized RdfS monomer (chain *B*) structure shown in black. (*d*) Overlaid backbone ribbon representation of RdfS_16–64_ for all *AlphaFold* models [coloured as in (*c*)] and each of the four RdfS chains (black). This region was highly similar between the models and each of the crystallographically determined RdfS chains (average r.m.s.d. between models of 1.0 Å: Supplementary Table S1).

**Figure 4:**
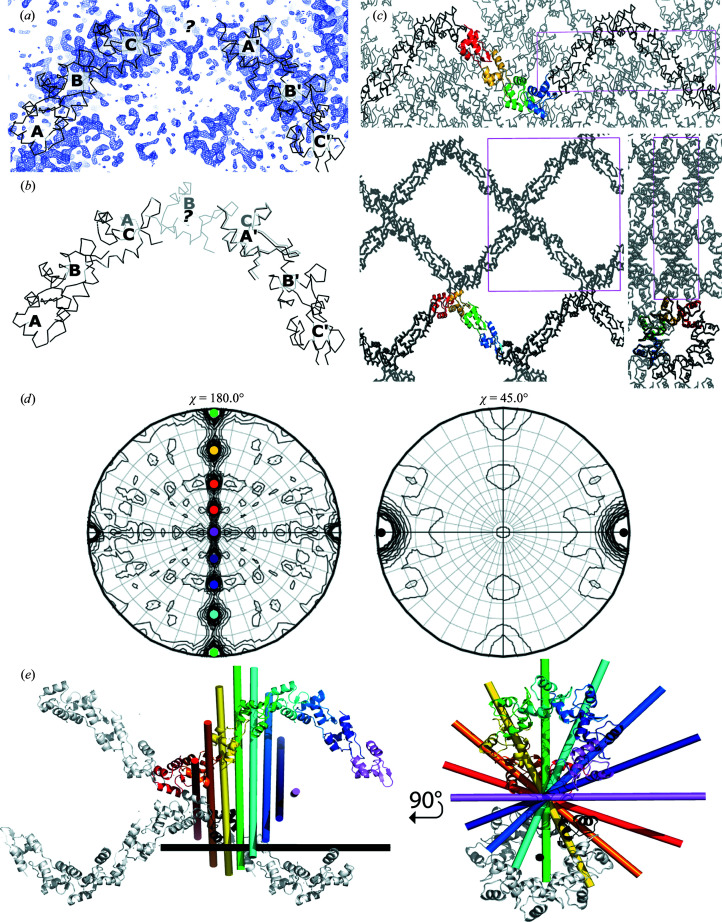
Molecular replacement of the RdfS crystal structure. (*a*) Initial molecular replacement located three RdfS monomers and a patch of unmodelled electron density. (*b*) Superposition of molecule *A* of the molecular-replacement solution on molecule *C* demonstrated a repetitive pattern of head-to-tail interacting monomers, allowing the placement of a fourth monomer. (*c*) Three orthogonal views of the final molecular-replacement solution, with four molecules in the asymmetric unit, result in a continuous left-handed superhelix with eight molecules per helical turn running through the crystal (pink box, unit cell). (*d*) The self-rotation function of RdfS indicates eight equally spaced twofold NCS axes (χ = 180°; four nonredundant axes related by a crystallographic twofold axis) and a strong eightfold NCS axis (χ = 45°). (*e*) Left: the rotational symmetry axes relating a monomer in one molecular superhelix (black) and eight monomers in two other asymmetric units (red, orange, yellow, green and cyan; blue, indigo and violet). The black bar represents the eightfold symmetry axis (χ = 45°) and the coloured bars represent each of the twofold symmetry axes (χ = 180°), as indicated on the self-rotation function. Right: an orthogonal view of the same set of molecules viewed along the eightfold symmetry axis (black).

Despite the lack of a close homologue to RdfS, the structure was solved by molecular replacement using *MOLREP* (Vagin & Teplyakov, 2010 ▸) from the *CCP*4 suite (Winn *et al.*, 2011 ▸). In the absence of structures of close homologues, *ab initio* predictions from *AlphaFold* were used as search models both individually and as a cluster of five models (Fig. 3 ▸). Initial molecular-replacement attempts used fully intact models to maximize the scattering power of the model, but failed most probably due to structural variation and disorder at the termini of the models (Fig. 3 ▸ *c* and Supplementary Table S1), an observation that was supported by disorder analysis with *DisEMBL* (version 1.5; Linding *et al.*, 2003 ▸) and the pLDDT plot of each model (Fig. 3 ▸ *a*). Manual investigation of the five individual *ab initio* models showed variability of the orientation of the N-terminal α-helix and the extended C-terminal tail, so a single truncated RdfS *AlphaFold* model (residues 16–64; RdfS_16–64_) was utilized in all further molecular-replacement attempts (Fig. 3 ▸ *b*). RdfS_16–64_ yielded promising results, with three protomers located with strong rotation-function and translation-function scores. Refinement of this model stalled, so we manually inspected the structure and electron density in *Coot*, with two notable observations: (i) the three molecules were arranged in a repeating head-to-tail manner (*i.e.* the interface of *A*:*B* was replicated at *B*:*C*) and (ii) there was notable electron density adjacent to molecule *C* (Fig. 4 ▸ *a*). Furthermore, structural alignment of the *A*:*B*:*C* ‘trimer’ on itself (with *A* superimposed on *C*; Fig. 4 ▸ *b*) placed molecule *B* in unmodelled electron density and molecule *C* directly on top of molecule *A*′ from an adjacent asymmetric unit. This was unlikely to be a coincidence and gave us confidence that the structure contains four molecules per asymmetric unit arranged in a head-to-tail fashion that continues throughout the crystal in one dimension, resulting in a protein polymer with eight molecules per helical turn. The refinement of four RdfS_16–64_ molecules (Fig. 3 ▸ *d*) proceeded as expected, with a substantial reduction of *R*_free_, and each copy was highly similar to the others in the asymmetric unit [average r.m.s.d. of 0.32 Å calculated using *PyMOL* (version 1.8; Schrödinger) with default cycle parameters; Supplementary Table S2]. A finalized structure refined to an *R*_work_ of 19.9% and an *R*_free_ of 24.6% validated using *MolProbity* (Chen *et al.*, 2010 ▸) has been deposited in the PDB (PDB entry 8dgl) and will be described in detail elsewhere.

## 4. Conclusion

The finalized RdfS monomer structure was determined to closely match a majority (∼57%) of the *ab initio* model as predicted by *AlphaFold*; it contains a single winged-helix–turn–helix domain, as is common among DNA-binding proteins (Aravind *et al.*, 2005 ▸; Brennan, 1993 ▸), and two additional helices at the N- and C-termini. We note that the 21 C-terminal amino acids of the protein are completely dis­ordered, as expected from their sequence PPEPGSDDDKGGSGSADEGARS. This represents 24% of the native protein sequence. Sequence comparisons reveal that this region of the protein is highly variable among RdfS homologues, likely due to the overlapping reading frames of *rdfS* and its downstream gene *traF* (Colombi *et al.*, 2021 ▸; Haskett *et al.*, 2016 ▸; Sullivan *et al.*, 2002 ▸), which suggests that it may not be the protein sequence of this region which is the trait under selection. It is possible, however, given the variability among relatives of the RdfS protein, that this extreme C-terminal region may regulate currently undiscovered species-specific functions of RdfS *in vivo*, such as aggregation or other forms of protein interaction, although this has not been experimentally tested. It is likely that the highly flexible nature of this C-terminal region, as partially described by the 6H-RdfS SAXS data detailed in this manuscript (Fig. 1 ▸), may have interfered with crystallization and contributed to the poor crystal quality.

Subsequent to this work, best practices for using *AlphaFold* models for molecular replacement have been developed (McCoy *et al.*, 2022 ▸). Analysis of the pLDDT confidence scores can be used to determine which residues to include from an *ab initio* model. Specifically, running *phenix.process_predicted_model* (Liebschner *et al.*, 2019 ▸) with the *AlphaFold* models of RdfS resulted in a trimmed model of residues 16–66, which closely matched our manually created model (Fig. 3 ▸ *b*). When manipulating *AlphaFold*-sourced structures, care should be taken to introduce realistic *B* factors into models in place of pLDDT scores, as implemented in the *MOLREP* ‘SURF Y’ command (Vagin & Teplyakov, 2010 ▸).

One of the axial views of the crystal structure (Fig. 4 ▸ *c*) reveals a highly porous crystal, which is commensurate with the observed solvent content of 71%. The porosity and limited contact between protomers may also explain the challenging crystallization and poor diffraction, as more loosely packed crystals typically diffract more weakly than more tightly packed crystals (Matthews, 1968 ▸, 1976 ▸; Podjarny *et al.*, 2002 ▸; Kantardjieff & Rupp, 2003 ▸).

RdfS proved a challenging protein to crystallize and solve the structure of, despite its relatively small size. It is likely that one cause of this difficulty is the observed propensity for the protein to form polymeric filaments. The biological significance of this quaternary structure has yet to be confirmed; however, the superficial similarity to the polymers formed by other structurally similar proteins such as BldC (Schumacher *et al.*, 2018 ▸; Dorman *et al.*, 2020 ▸) and Xis (Abbani *et al.*, 2007 ▸) may suggest a role for cooperative DNA binding in the regulation of ICE excision, the activation of conjugative transfer and transcriptional regulation. Further investigations on the biological implications of the quaternary structure of RdfS, along with implicit discussions on protein–nucleic acid interactions, will be presented in the future.

## Supplementary Material

PDB reference: RdfS, 8dgl

SASBDB reference: RdfS, SASDPK4

Supplementary Table and Figures. DOI: 10.1107/S2059798322008579/rr5226sup1.pdf

## Figures and Tables

**Table 1:** SAXS data-collection and analysis statistics for 6H-RdfS

Data collection	
Instrument	SAXS/WAXS, Australian Synchrotron
Strategy	SEC-SY-SAXS
SEC column	Superdex 200 Increase 5/150 GL
Beam geometry (µm)	22
Wavelength (Å)	1.0332
*q* range (Å^−1^)	0.004–0.382
Exposure per frame (s)	5.00
Flow rate (ml min^−1^)	0.3
Concentration (mg ml^−1^)	6.8 (at injection)
Temperature (K)	298
Guinier parameters	
*I*(0) (cm^−1^)	0.011 ± 0.000
*R*_g_ (Å)	22.20 ± 0.21
Guinier range (*q* ^2^)	0.00001–0.00337

**Table 2:** Crystallograpic data-collection and processing statistics for RdfS Values in parentheses are for the outer shell.

Data collection	
Space group	*P*2_1_2_1_2_1_
*a*, *b*, *c* (Å)	35.25, 119.20, 123.40
Mosaicity (°)	0.1
Resolution range (Å)	42.87–2.45 (2.55–2.45)
Total No. of reflections	132047 (14108)
No. of unique reflections	19949 (2118)
Completeness (%)	99.4 (94.7)
Multiplicity	6.6 (6.7)
〈*I*/σ(*I*)〉	9.0 (2.4)
CC_1/2_	0.993 (0.744)
*R*_meas_	0.159 (0.935)
Refinement parameters	
Overall *B* factor from Wilson plot (Å^2^)	31.1
Average *B* factors (Å^2^)	
Protein	44.5
Ion	68.0
Ligand	58.8
Water	46.2
Ramachandran plot	
Most favoured	261 [100%]
Allowed	1 [0%]
R.m.s.d., bond lengths (Å)	0.008
R.m.s.d., bond angles (°)	1.159
Total reflections	18962 (1796)
Free reflections	933 [4.7%] (78 [4.2%])
Final *R*_cryst_	0.199 (0.265)
Final *R*_free_	0.246 (0.321)

## References

[bb1] Abbani, M. A., Papagiannis, C. V., Sam, M. D., Cascio, D., Johnson, R. C. & Clubb, R. T. (2007). *Proc. Natl Acad. Sci. USA*, **104**, 2109–2114. 10.1073/pnas.0607820104 PMC1893000 17287355

[bb2] Afonine, P. V., Grosse-Kunstleve, R. W., Echols, N., Headd, J. J., Moriarty, N. W., Mustyakimov, M., Terwilliger, T. C., Urzhumtsev, A., Zwart, P. H. & Adams, P. D. (2012). *Acta Cryst.* D**68**, 352–367. 10.1107/S0907444912001308 PMC3322595 22505256

[bb3] Ahlgren-Berg, A., Cardoso-Palacios, C., Eriksson, J. M., Mandali, S., Sehlén, W., Sylwan, L. & Haggård-Ljungquist, E. (2009). *Virology*, **385**, 303–312. 10.1016/j.virol.2008.12.002 19150106

[bb4] Aragão, D., Aishima, J., Cherukuvada, H., Clarken, R., Clift, M., Cowieson, N. P., Ericsson, D. J., Gee, C. L., Macedo, S., Mudie, N., Panjikar, S., Price, J. R., Riboldi-Tunnicliffe, A., Rostan, R., Williamson, R. & Caradoc-Davies, T. T. (2018). *J. Synchrotron Rad.* **25**, 885–891. 10.1107/S1600577518003120 PMC5929359 29714201

[bb5] Aravind, L., Anantharaman, V., Balaji, S., Babu, M. M. & Iyer, L. M. (2005). *FEMS Microbiol. Rev.* **29**, 231–262. 10.1016/j.femsre.2004.12.008 15808743

[bb6] Berman, H. M., Westbrook, J., Feng, Z., Gilliland, G., Bhat, T. N., Weissig, H., Shindyalov, I. N. & Bourne, P. E. (2000). *Nucleic Acids Res.* **28**, 235–242. 10.1093/nar/28.1.235 PMC102472 10592235

[bb7] Bernadó, P., Mylonas, E., Petoukhov, M. V., Blackledge, M. & Svergun, D. I. (2007). *J. Am. Chem. Soc.* **129**, 5656–5664. 10.1021/ja069124n 17411046

[bb8] Besl, P. J. & McKay, N. D. (1992). *IEEE Trans. Pattern Anal. Mach. Intell.* **14**, 239–256.

[bb9] Bizien, T., Durand, D., Roblina, P., Thureau, A., Vachette, P. & Pérez, J. (2016). *Protein Pept. Lett.* **23**, 217–231. 10.2174/0929866523666160106153655 26732245

[bb10] Brennan, R. G. (1993). *Cell*, **74**, 773–776. 10.1016/0092-8674(93)90456-z 8374950

[bb11] Broennimann, C., Eikenberry, E. F., Henrich, B., Horisberger, R., Huelsen, G., Pohl, E., Schmitt, B., Schulze-Briese, C., Suzuki, M., Tomizaki, T., Toyokawa, H. & Wagner, A. (2006). *J. Synchrotron Rad.* **13**, 120–130. 10.1107/S0909049505038665 16495612

[bb12] Brünger, A. T. (1992). *Nature*, **355**, 472–475. 10.1038/355472a0 18481394

[bb13] Casanas, A., Warshamanage, R., Finke, A. D., Panepucci, E., Olieric, V., Nöll, A., Tampé, R., Brandstetter, S., Förster, A., Mueller, M., Schulze-Briese, C., Bunk, O. & Wang, M. (2016). *Acta Cryst.* D**72**, 1036–1048. 10.1107/S2059798316012304 PMC5013597 27599736

[bb14] Chen, V. B., Arendall, W. B., Headd, J. J., Keedy, D. A., Immormino, R. M., Kapral, G. J., Murray, L. W., Richardson, J. S. & Richardson, D. C. (2010). *Acta Cryst.* D**66**, 12–21. 10.1107/S0907444909042073 PMC2803126 20057044

[bb15] Colombi, E., Perry, B. J., Sullivan, J. T., Bekuma, A. A., Terpolilli, J. J., Ronson, C. W. & Ramsay, J. P. (2021). *Microb. Genomics*, **7**, e000657. 10.1099/mgen.0.000657 PMC8627217 34605762

[bb16] Dodd, I. B., Kalionis, B. & Egan, J. B. (1990). *J. Mol. Biol.* **214**, 27–37. 10.1016/0022-2836(90)90144-B 2370665

[bb17] Dorman, C. J., Schumacher, M. A., Bush, M. J., Brennan, R. G. & Buttner, M. J. (2020). *Curr. Opin. Microbiol.* **55**, 26–33. 10.1016/j.mib.2020.01.019 PMC8048100 32120333

[bb18] Dümmler, A., Lawrence, A. & de Marco, A. (2005). *Microb. Cell Fact.* **4**, 34. 10.1186/1475-2859-4-34 PMC1326211 16351710

[bb19] Durand, D., Vivès, C., Cannella, D., Pérez, J., Pebay-Peyroula, E., Vachette, P. & Fieschi, F. (2010). *J. Struct. Biol.* **169**, 45–53. 10.1016/j.jsb.2009.08.009 19723583

[bb20] Emsley, P., Lohkamp, B., Scott, W. G. & Cowtan, K. (2010). *Acta Cryst.* D**66**, 486–501. 10.1107/S0907444910007493 PMC2852313 20383002

[bb21] Esposito, D. & Scocca, J. J. (1997). *Nucleic Acids Res.* **25**, 3605–3614. 10.1093/nar/25.18.3605 PMC146934 9278480

[bb22] Evans, P. R. & Murshudov, G. N. (2013). *Acta Cryst.* D**69**, 1204–1214. 10.1107/S0907444913000061 PMC3689523 23793146

[bb23] Fischer, H., de Oliveira Neto, M., Napolitano, H. B., Polikarpov, I. & Craievich, A. F. (2010). *J. Appl. Cryst.* **43**, 101–109.

[bb24] Franke, D. & Svergun, D. I. (2009). *J. Appl. Cryst.* **42**, 342–346. 10.1107/S0021889809000338 PMC5023043 27630371

[bb25] Groth, A. C. & Calos, M. P. (2004). *J. Mol. Biol.* **335**, 667–678. 10.1016/j.jmb.2003.09.082 14687564

[bb26] Gully, B. S., Cowieson, N., Stanley, W. A., Shearston, K., Small, I. D., Barkan, A. & Bond, C. S. (2015). *Nucleic Acids Res.* **43**, 1918–1926. 10.1093/nar/gkv027 PMC4330388 25609698

[bb27] Haas, D. J. & Rossmann, M. G. (1970). *Acta Cryst.* B**26**, 998–1004. 10.1107/s0567740870003485 5536135

[bb28] Hajizadeh, N. R., Franke, D., Jeffries, C. M. & Svergun, D. I. (2018). *Sci. Rep.* **8**, 7204. 10.1038/s41598-018-25355-2 PMC5940760 29739979

[bb29] Haskett, T. L., Ramsay, J. P., Bekuma, A. A., Sullivan, J. T., O’Hara, G. W. & Terpolilli, J. J. (2017). *Plasmid*, **92**, 30–36. 10.1016/j.plasmid.2017.06.001 28669811

[bb30] Haskett, T. L., Terpolilli, J. J., Bekuma, A., O’Hara, G. W., Sullivan, J. T., Wang, P., Ronson, C. W. & Ramsay, J. P. (2016). *Proc. Natl Acad. Sci. USA*, **113**, 12268–12273. 10.1073/pnas.1613358113 PMC5087020 27733511

[bb31] Haskett, T. L., Terpolilli, J. J., Ramachandran, V. K., Verdonk, C. J., Poole, P. S., O’Hara, G. W. & Ramsay, J. P. (2018). *PLoS Genet.* **14**, e1007292. 10.1371/journal.pgen.1007292 PMC5882170 29565971

[bb32] Henderson, R. (1990). *Proc. R. Soc. Lond. B*, **241**, 6–8.

[bb33] Jumper, J., Evans, R., Pritzel, A., Green, T., Figurnov, M., Ronneberger, O., Tunyasuvunakool, K., Bates, R., Žídek, A., Potapenko, A., Bridgland, A., Meyer, C., Kohl, S. A. A., Ballard, A. J., Cowie, A., Romera-Paredes, B., Nikolov, S., Jain, R., Adler, J., Back, T., Petersen, S., Reiman, D., Clancy, E., Zielinski, M., Steinegger, M., Pacholska, M., Berghammer, T., Bodenstein, S., Silver, D., Vinyals, O., Senior, A. W., Kavukcuoglu, K., Kohli, P. & Hassabis, D. (2021). *Nature*, **596**, 583–589. 10.1038/s41586-021-03819-2 PMC8371605 34265844

[bb34] Kabsch, W. (2010). *Acta Cryst.* D**66**, 125–132. 10.1107/S0907444909047337 PMC2815665 20124692

[bb35] Kantardjieff, K. A. & Rupp, B. (2003). *Protein Sci.* **12**, 1865–1871. 10.1110/ps.0350503 PMC2323984 12930986

[bb36] Kirby, N. M., Mudie, S. T., Hawley, A. M., Cookson, D. J., Mertens, H. D. T., Cowieson, N. & Samardzic-Boban, V. (2013). *J. Appl. Cryst.* **46**, 1670–1680.

[bb37] Konarev, P. V., Volkov, V. V., Sokolova, A. V., Koch, M. H. J. & Svergun, D. I. (2003). *J. Appl. Cryst.* **36**, 1277–1282.

[bb38] Lewis, J. A. & Hatfull, G. F. (2001). *Nucleic Acids Res.* **29**, 2205–2216. 10.1093/nar/29.11.2205 PMC55702 11376138

[bb39] Liebschner, D., Afonine, P. V., Baker, M. L., Bunkóczi, G., Chen, V. B., Croll, T. I., Hintze, B., Hung, L.-W., Jain, S., McCoy, A. J., Moriarty, N. W., Oeffner, R. D., Poon, B. K., Prisant, M. G., Read, R. J., Richardson, J. S., Richardson, D. C., Sammito, M. D., Sobolev, O. V., Stockwell, D. H., Terwilliger, T. C., Urzhumtsev, A. G., Videau, L. L., Williams, C. J. & Adams, P. D. (2019). *Acta Cryst.* D**75**, 861–877.

[bb40] Linding, R., Jensen, L. J., Diella, F., Bork, P., Gibson, T. J. & Russell, R. B. (2003). *Structure*, **11**, 1453–1459. 10.1016/j.str.2003.10.002 14604535

[bb41] Lundqvist, B. & Bertani, G. (1984). *J. Mol. Biol.* **178**, 629–651. 10.1016/0022-2836(84)90242-0 6492160

[bb50] Manalastas-Cantos, K., Konarev, P. V., Hajizadeh, N. R., Kikhney, A. G., Petoukhov, M. V., Molodenskiy, D. S., Panjkovich, A., Mertens, H. D. T., Gruzinov, A., Borges, C., Jeffries, C. M., Svergun, D. I. & Franke, D. (2021). *J. Appl. Cryst.* **54**, 343–355. 10.1107/S1600576720013412 PMC7941305 33833657

[bb43] Matthews, B. W. (1968). *J. Mol. Biol.* **33**, 491–497. 10.1016/0022-2836(68)90205-2 5700707

[bb42] Matthews, B. W. (1976). *Annu. Rev. Phys. Chem.* **27**, 493–523.

[bb44] McCoy, A. J., Sammito, M. D. & Read, R. J. (2022). *Acta Cryst.* D**78**, 1–13. 10.1107/S2059798321012122 PMC8725160 34981757

[bb45] McPhillips, T. M., McPhillips, S. E., Chiu, H.-J., Cohen, A. E., Deacon, A. M., Ellis, P. J., Garman, E., Gonzalez, A., Sauter, N. K., Phizackerley, R. P., Soltis, S. M. & Kuhn, P. (2002). *J. Synchrotron Rad.* **9**, 401–406. 10.1107/s0909049502015170 12409628

[bb46] Mirdita, M., Schütze, K., Moriwaki, Y., Heo, L., Ovchinnikov, S. & Steinegger, M. (2022). *Nat. Methods*, **19**, 679–682. 10.1038/s41592-022-01488-1 PMC9184281 35637307

[bb47] Murshudov, G. N., Skubák, P., Lebedev, A. A., Pannu, N. S., Steiner, R. A., Nicholls, R. A., Winn, M. D., Long, F. & Vagin, A. A. (2011). *Acta Cryst.* D**67**, 355–367. 10.1107/S0907444911001314 PMC3069751 21460454

[bb48] Necci, M., Piovesan, D., Clementel, D., Dosztányi, Z. & Tosatto, S. C. E. (2021). *Bioinformatics*, **36**, 5533–5534. 10.1093/bioinformatics/btaa1045 33325498

[bb49] Newman, J., Egan, D., Walter, T. S., Meged, R., Berry, I., Ben Jelloul, M., Sussman, J. L., Stuart, D. I. & Perrakis, A. (2005). *Acta Cryst.* D**61**, 1426–1431. 10.1107/S0907444905024984 16204897

[bb51] Podjarny, A., Howard, E., Mitschler, A., Chevrier, B., Lecomte, C., Guillot, B., Pichon-Pesme, V. & Jelsch, C. (2002). *Europhys. News*, **33**, 113–117.

[bb52] Radman-Livaja, M., Biswas, T., Mierke, D. & Landy, A. (2005). *Proc. Natl Acad. Sci. USA*, **102**, 3913–3920. 10.1073/pnas.0500844102 PMC554831 15753294

[bb54] Ramsay, J. P., Bastholm, T. R., Verdonk, C. J., Tambalo, D. D., Sullivan, J., Harold, L., Panganiban, B. A., Colombi, E., Perry, B., Jowsey, W., Morris, C., Hynes, M., Bond, C., Cameron, A. D. S., Yost, C. & Ronson, C. (2021). *Nucleic Acids Res.* **50**, 975–988. 10.1093/nar/gkab1217 PMC8789080 34904658

[bb55] Ramsay, J. P., Sullivan, J. T., Stuart, G. S., Lamont, I. L. & Ronson, C. W. (2006). *Mol. Microbiol.* **62**, 723–734. 10.1111/j.1365-2958.2006.05396.x 17076666

[bb53] Ramsay, J. P., Tester, L. G. L., Major, A. S., Sullivan, J. T., Edgar, C. D., Kleffmann, T., Patterson-House, J. R., Hall, D. A., Tate, W. P., Hynes, M. F. & Ronson, C. W. (2015). *Proc. Natl Acad. Sci. USA*, **112**, 4104–4109. 10.1073/pnas.1501574112 PMC4386382 25787256

[bb56] Saha, S., Haggård-Ljungquist, E. & Nordström, K. (1987). *EMBO J.* **6**, 3191–3199. 10.1002/j.1460-2075.1987.tb02631.x PMC553762 2826134

[bb57] Sam, M. D., Cascio, D., Johnson, R. C. & Clubb, R. T. (2004). *J. Mol. Biol.* **338**, 229–240. 10.1016/j.jmb.2004.02.053 15066428

[bb59] Schumacher, M. A., den Hengst, C. D., Bush, M. J., Le, T. B. K., Tran, N. T., Chandra, G., Zeng, W., Travis, B., Brennan, R. G. & Buttner, M. J. (2018). *Nat. Commun.* **9**, 1139. 10.1038/s41467-018-03576-3 PMC5859096 29556010

[bb60] Semenyuk, A. V. & Svergun, D. I. (1991). *J. Appl. Cryst.* **24**, 537–540.

[bb61] Steinegger, M., Meier, M., Mirdita, M., Vöhringer, H., Haunsberger, S. J. & Söding, J. (2019). *BMC Bioinformatics*, **20**, 473. 10.1186/s12859-019-3019-7 PMC6744700 31521110

[bb62] Steinegger, M. & Söding, J. (2017). *Nat. Biotechnol.* **35**, 1026–1028. 10.1038/nbt.3988 29035372

[bb63] Sullivan, J. & Ronson, C. (1998). *Proc. Natl Acad. Sci. USA*, **95**, 5145–5149. 10.1073/pnas.95.9.5145 PMC20228 9560243

[bb64] Sullivan, J. T., Trzebiatowski, J. R., Cruickshank, R. W., Gouzy, J., Brown, S. D., Elliot, R. M., Fleetwood, D. J., McCallum, N. G., Rossbach, U., Stuart, G. S., Weaver, J. E., Webby, R. J., de Bruijn, F. J. & Ronson, C. W. (2002). *J. Bacteriol.* **184**, 3086–3095. 10.1128/JB.184.11.3086-3095.2002 PMC135072 12003951

[bb65] Svergun, D. I. (1999). *Biophys. J.* **76**, 2879–2886. 10.1016/S0006-3495(99)77443-6 PMC1300260 10354416

[bb66] Trewhella, J., Duff, A. P., Durand, D., Gabel, F., Guss, J. M., Hendrickson, W. A., Hura, G. L., Jacques, D. A., Kirby, N. M., Kwan, A. H., Pérez, J., Pollack, L., Ryan, T. M., Sali, A., Schneidman-Duhovny, D., Schwede, T., Svergun, D. I., Sugiyama, M., Tainer, J. A., Vachette, P., Westbrook, J. & Whitten, A. E. (2017). *Acta Cryst.* D**73**, 710–728. 10.1107/S2059798317011597 PMC5586245 28876235

[bb67] Tria, G., Mertens, H. D. T., Kachala, M. & Svergun, D. I. (2015). *IUCrJ*, **2**, 207–217. 10.1107/S205225251500202X PMC4392415 25866658

[bb68] Usón, I., Pohl, E., Schneider, T. R., Dauter, Z., Schmidt, A., Fritz, H. J. & Sheldrick, G. M. (1999). *Acta Cryst.* D**55**, 1158–1167. 10.1107/s0907444999003972 10329778

[bb69] Vagin, A. & Teplyakov, A. (2010). *Acta Cryst.* D**66**, 22–25. 10.1107/S0907444909042589 20057045

[bb70] Verdonk, C. J., Sullivan, J. T., Williman, K. M., Nicholson, L., Bastholm, T. R., Hynes, M. F., Ronson, C. W., Bond, C. S. & Ramsay, J. P. (2019). *Plasmid*, **104**, 102416. 10.1016/j.plasmid.2019.102416 31078551

[bb71] Volkov, V. V. & Svergun, D. I. (2003). *J. Appl. Cryst.* **36**, 860–864.

[bb72] Wilkins, M. R., Gasteiger, E., Bairoch, A., Sanchez, J. C., Williams, K. L., Appel, R. D. & Hochstrasser, D. F. (1999). *Methods Mol. Biol.* **112**, 531–552. 10.1385/1-59259-584-7:531 10027275

[bb73] Winn, M. D., Ballard, C. C., Cowtan, K. D., Dodson, E. J., Emsley, P., Evans, P. R., Keegan, R. M., Krissinel, E. B., Leslie, A. G. W., McCoy, A., McNicholas, S. J., Murshudov, G. N., Pannu, N. S., Potterton, E. A., Powell, H. R., Read, R. J., Vagin, A. & Wilson, K. S. (2011). *Acta Cryst.* D**67**, 235–242. 10.1107/S0907444910045749 PMC3069738 21460441

