# Supplementary material for: Crystallographic and X-ray scattering study of RdfS, a recombination directionality factor from an integrative and conjugative element
Source: Acta Crystallogr D Struct Biol. 2022 Sep 27;78(Pt 10):1210–20. doi: 10.1107/S2059798322008579 (PMC9527761; doi:10.1107/S2059798322008579)
Supplement: Supplementary file 1 [file d-78-01210-sup1.pdf]

STRUCTURAL
BIOLOGY

Volume 78 (2022)

Supporting information for article:

Crystallographic and X-ray scattering study of RdfS, a recombination directionality factor from an integrative and conjugative element

Callum J. Verdonk, Andrew C. Marshall, Joshua P. Ramsay and Charles S. Bond

Table S1 The root-mean-square deviation (RMSD) of atomic positions between AlphaFold-generated models used as queries in Molecular Replacement described in section 3.3. Number of atoms shown in brackets. RMSD between alpha-carbons (Cα), and outliers-removed also shown. All values as calculated by Pymol *align* function.

**Models compared**	**RMSD (Å)**	**RMSD (all atoms) (Å)**	**RMSD Cα/Cα no outliers (Å)**
Model 1:Model 2	0.974 (523)	4.773 (690)	0.871 (68)/4.397 (89)
Model 1:Model 3	1.055 (475)	6.059 (690)	0.938 (60)/5.510 (89)
Model 1:Model 4	0.746 (499)	3.648 (690)	0.600 (62)/3.023 (89)
Model 1:Model 5	1.207 (513)	5.324 (690)	1.203 (67)/4.797 (89)
Model 2:Model 3	0.737 (481)	7.167 (690)	0.701 (61)/6.705 (89)
Model 2:Model 4	0.940 (508)	5.827 (690)	0.892 (67)/5.313 (89)
Model 2:Model 5	1.003 (494)	4.227 (690)	0.820 (64)/3.636 (89)
Model 3:Model 4	1.261 (516)	3.665 (690)	1.284 (71)/3.255 (89)
Model 3:Model 5	1.229 (469)	6.509 (690)	1.598 (66)/5.852 (89)
Model 4:Model 5	0.913 (484)	4.741 (690)	1.010 (64)/4.449 (89)
Average	1.007	5.194	0.992/4.694

Table S2 RMSD for each individual chain (monomer) of RdfS in the asymmetric unit. Number of atoms compared in brackets. All values as calculated by Pymol *align* function.

**Chains compared**	**RMSD (Å)**	**RMSD (all atoms) (Å)**	**RMSD Cα/Cα no outliers (Å)**
A:B	0.236 (421)	0.993 (536)	0.118 (54)/0.601 (67)
A:C	0.388 (434)	1.020 (535)	0.356 (58)/0.602 (67)
A:D	0.234 (424)	1.027 (535)	0.205 (59)/0.579 (67)
B:C	0.311 (426)	1.044 (535)	0.248 (56)/0.404 (67)
B:D	0.300 (418)	1.098 (535)	0.241 (56)/0.457 (67)
C:D	0.441 (441)	0.927 (535)	0.388 (58)/0.537 (67)
Average	0.318	1.02	0.260/0.530

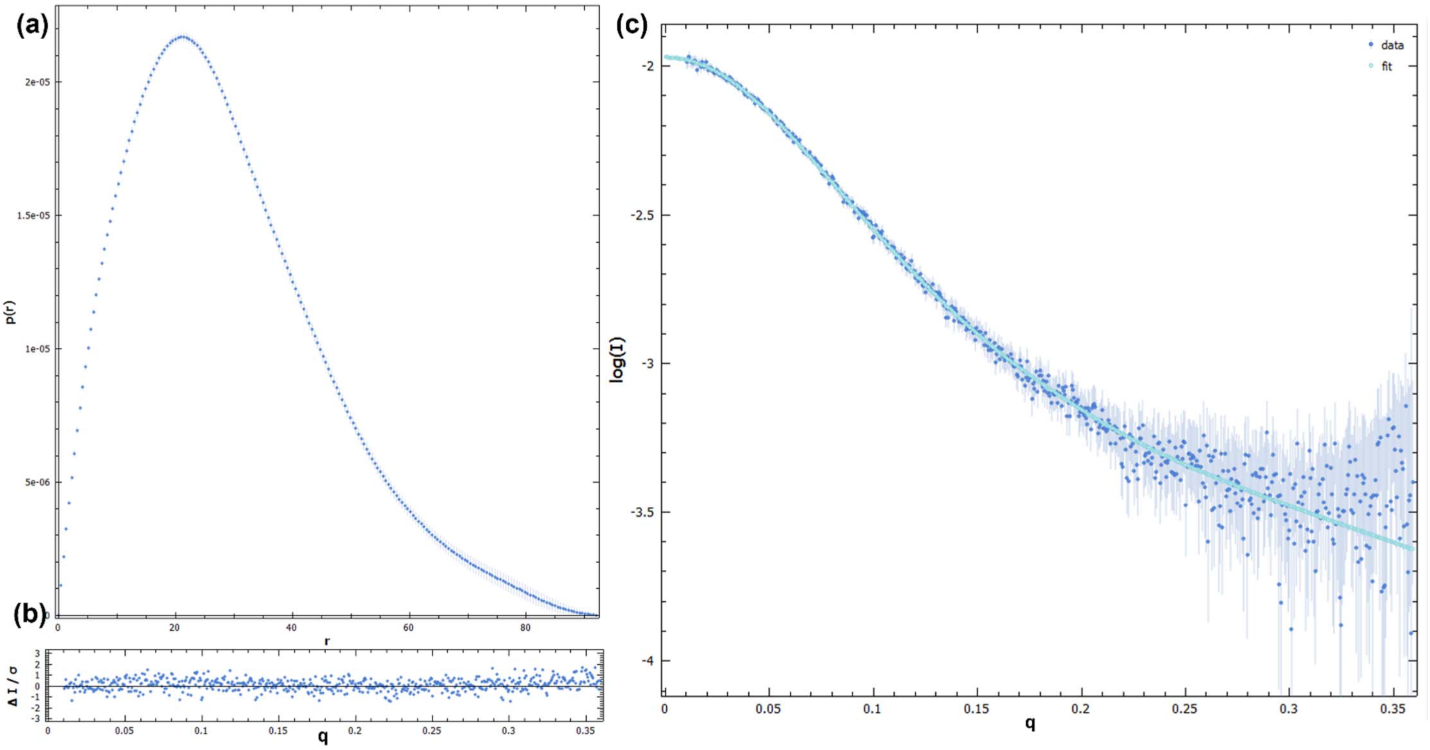

Figure S1 Scattering plot and model fit for pair-distribution P(r) function (inverse Fourier transform of *I*(q)) using GNOM. **(a)** D_{max} plot suggesting 6H-RdfS has a rather elongated shape of approx. 92 Å. Note the smooth approach to 0 for r. **(b)** accompanying normalised residual fit for P(r). **(c)** Graphical representation of P(r) fit (light blue line) into logarithmic scattering data (dark blue points).

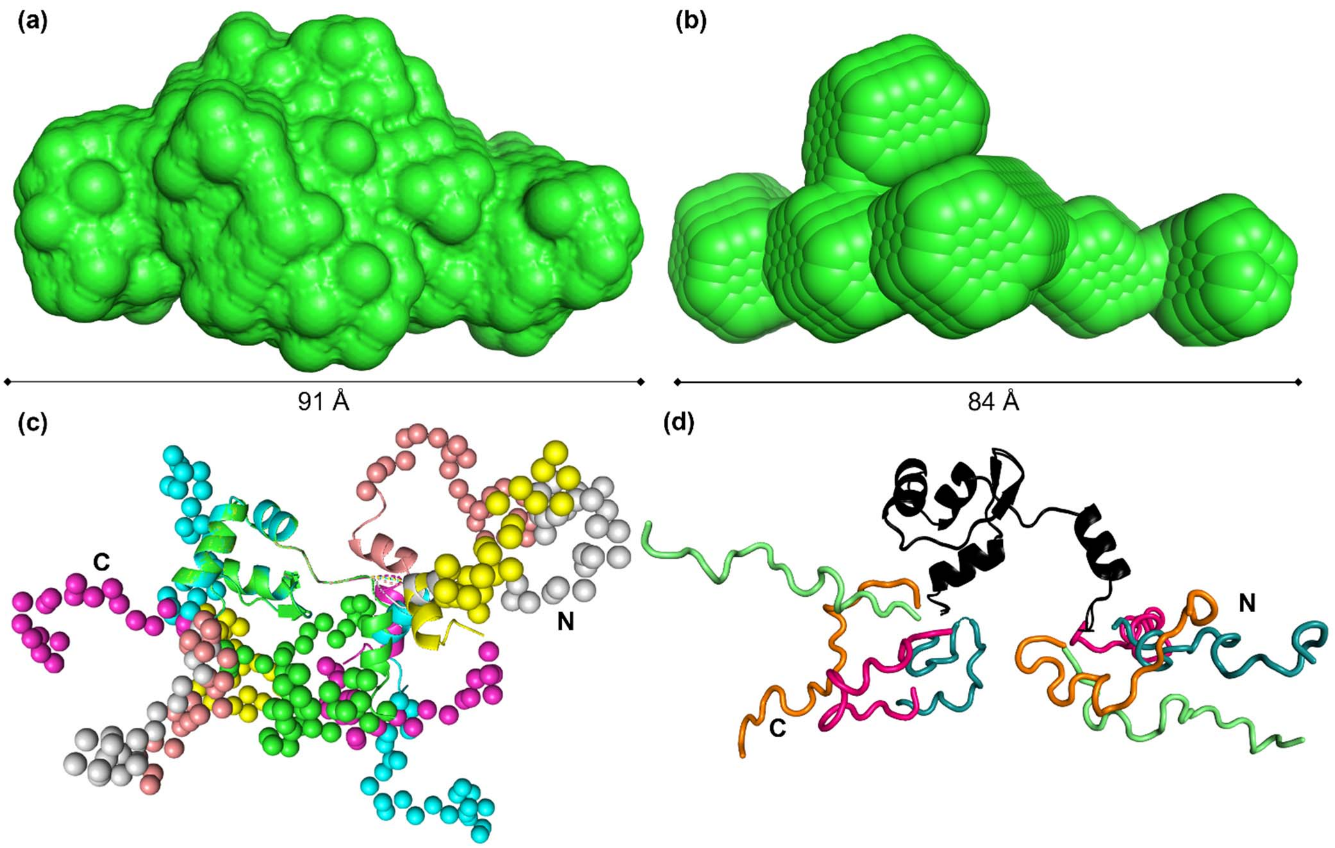

Figure S2 Various models of 6H-RdfS based on SAXS scattering data. **(a)** *Ab initio* DAMAVER-refined global bead model showing the scattering envelope of 6H-RdfS. The distance estimate of 91 Å aligns closely with the D_{max} calculated in Sup Fig 2. All 11 DAMMIN-generate models were include in DAMAVER calculations, suggesting each model converged toward a similar shape. **(b)** DAMMIF-generated *ab initio* bead model showing 6H-RdfS scattering envelop. Note the similarities to (a) and Fig 1e. **(c)** Six EOM-generated model of 6H-RdfS using the X-ray structure of RdfS: with the wHTH domain fixed and N-terminal helix allocated as flexible. This model is the same as Fig 1f, with the addition of the dummy residues allocated to the N-terminal (25 residues total: 6H tag, flexible linker, TEV-site) and undetermined 21-residues from the X-ray structure. **(d)** Four CRYSOL-generated models of 6H-RdfS utilising the entire X-ray structure of RdfS as a template model defined as rigid. Note the flexibility of the N- and C- termini in both (c) and (d) models.

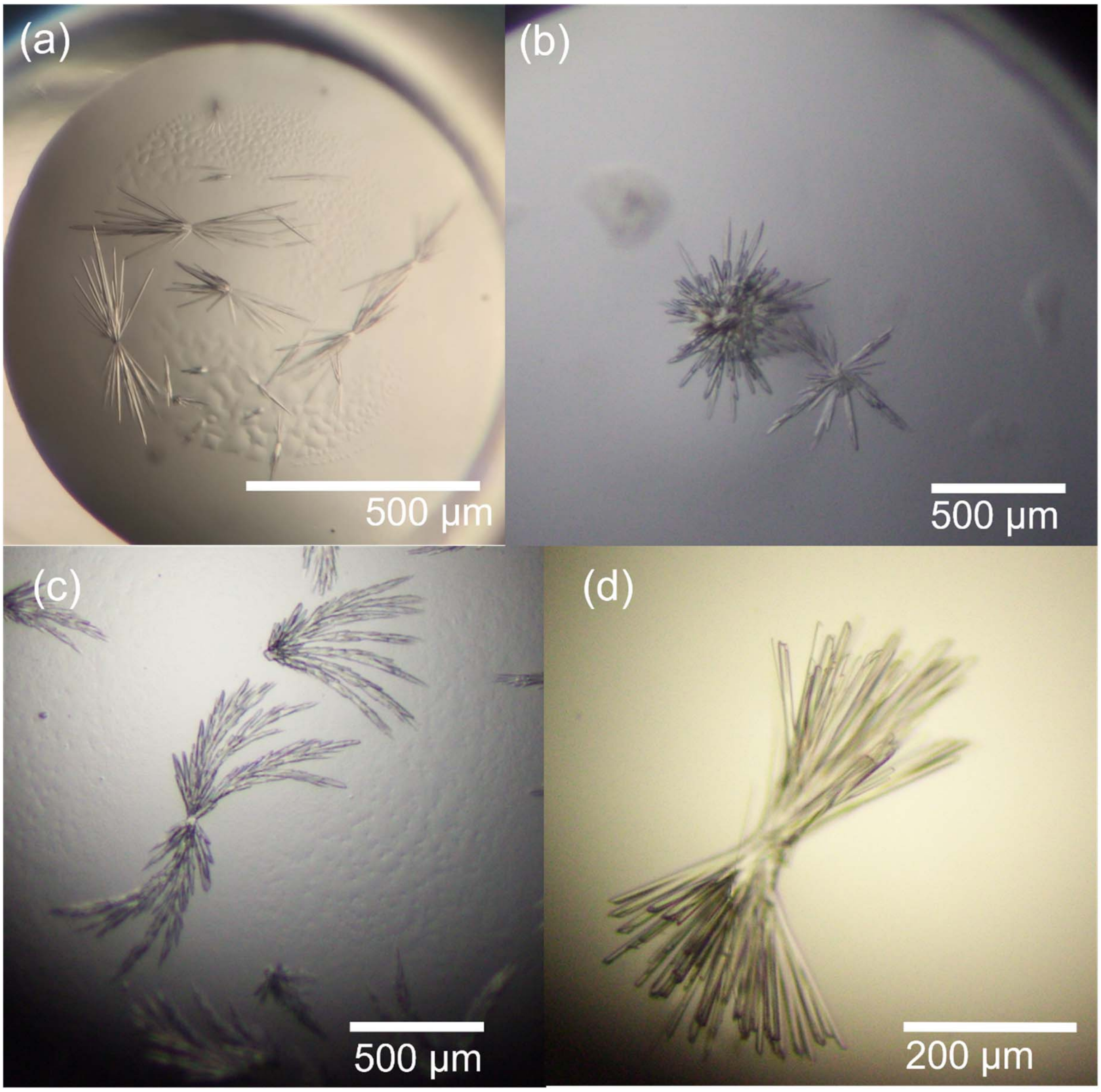

Figure S3 Additional representative crystals of RdfS. **(a)** Index-screen condition showing typical RdfS needle-like crystals (Index screen condition G6). **(b)** ProPlex screen condition showing large needle star-like multinuclear RdfS crystals (ProPlex condition 38). **(c)** Condition chosen for additive screen optimisation, a variant of condition 38 in ProPlex screen (drop condition: 0.1 M MES pH 6.5, 8% *w/v* PEG 5000 MME, 10% *v/v* 1-propanol). **(d)** Variant of final condition (drop condition: 4 µL condition from (c) + 1 µL 0.05 M MES pH 6.5, 4% *w/v* PEG 5000 MME, 5% *v/v* 1-propanol, 0.1 M sodium citrate tribasic dehydrate. Buffer reservoir was 100 µL 0.05 M MES pH 6.5, 4% *w/v* PEG 5000 MME, 5% *v/v* 1-propanol, 0.1 M sodium citrate tribasic dehydrate). No significant diffraction was seen for any of these crystals (none better than 10 Å).
